# The Use of Enhanced Recovery After Surgery Protocols and Sugammadex in a Friedreich Ataxia Patient Who Underwent Robotic Surgery: A Case Report of a Patient Who Required No Postoperative Opioids and Was Discharged Home Earlier Than Anticipated

**DOI:** 10.7759/cureus.29590

**Published:** 2022-09-26

**Authors:** Lori P Russo, Daniel Haddad, Daniel Bauman, Mina M Fam

**Affiliations:** 1 Anesthesiology, North American Partners in Anesthesia (NAPA), Hackensack Meridian Ocean University Medical Center, Brick, USA; 2 Anesthesia, Rowan University School of Osteopathic Medicine, Stratford, USA; 3 Urology, Hackensack Meridian Ocean University Medical Center, Brick, USA

**Keywords:** friedreich ataxia, multimodal pain management, neuromuscular diseases, urology and oncology, general anesthesia practice, quadratus lumborum block, sugammadex, robotic partial nephrectomy, robotic surgery procedures, enhanced recovery after surgery

## Abstract

Robotic surgery has shown to have numerous benefits over traditional and laparoscopic surgery, namely, superior precision and improved recovery with shorter hospital stays. However, robotic surgery also presents several issues, including hemodynamic changes related to positioning and the use of pneumoperitoneum. These matters can be problematic in patients with neuromuscular conditions such as Friedreich ataxia (FRDA). Due to a baseline weakened musculature and a higher prevalence of cardiac disease and scoliosis, patients with FRDA may not be as likely to tolerate the cardiopulmonary physiologic changes associated with robotic surgery. Additionally, positioning for robotic surgery can be challenging in FRDA patients who have progressed to spasticity and contractures. To the best of our knowledge, there are no case reports of approaches specifically discussing anesthesia management for robotic surgery in the FRDA patient population.

Anesthesia in general must be carefully planned in FRDA patients to allow for the best possible recovery and minimize complications. Due to the underlying neuromuscular compromise seen in these patients, their ability to recover from the pharmacologic and physiologic changes associated with anesthesia can be more difficult. They are prone to sensitivity to opioids, sedatives, and neuromuscular blocking agents (NMBAs) and are less likely to tolerate hemodynamic changes. Our review revealed no literature to suggest the routine use of Enhanced Recovery After Surgery (ERAS) protocols in FRDA patients or in patients with neuromuscular disease in general.

The use of sugammadex has also been shown to be safe, and literature suggests superiority in both the general population and those with neuromuscular conditions. Our understanding is that there is very limited literature in regard to the safe use of sugammadex in FRDA patients.

## Introduction

Friedreich ataxia (FRDA) was originally described by Nikolaus Friedreich in 1863 and is classified as spinocerebellar ataxia. It is inherited in an autosomal recessive manner and, in 1996, was found to be associated with abnormally elevated trinucleotide GAA repeats. This trinucleotide expansion results in the silencing of the FXN gene and the subsequent pathological suppression of frataxin protein production. While it is the most common genetically acquired ataxia in Europe, the prevalence of the disease varies by region and ranges between one in 20,000 and one in 250,000. The pathophysiology includes multisystem dysfunction of central and peripheral nervous systems in addition to the myocardium and pancreas. An increased incidence of diabetes mellitus, cardiomyopathy, and respiratory dysfunction is commonly seen. The typical presentation includes gait and limb ataxia, dysarthria, and reduction or loss of lower extremity reflexes. Symptom onset typically begins in the early teenage years with mixed gait and limb ataxia, with many patients becoming wheelchair-bound by the time they enter their 30s. Spasticity is a common feature, and contractures often follow. Dysphagia is frequently seen in the FRDA population and is typically progressive. Adequate nutrition may require the placement of nasogastric tubes or gastrostomy. Optic neuropathy develops in a fraction of patients and can result in blindness, and sensorineural loss of hearing has been noted in the literature. Frequency of urination and urgency with an overactive bladder are seen in a substantial number of FRDA patients and are thought to be related to pyramidal dysfunction. Bowel motility can be affected and may manifest as either incontinence or constipation. The incidence of obstructive sleep apnea (OSA) is higher in patients with a diagnosis of FRDA than in the general population. It is believed that the duration and severity of the disease play a role in the development of OSA [1].

In patients with FRDA, special attention must be given to the preoperative evaluation of cardiovascular and respiratory function. For FRDA patients undergoing major surgery, it is recommended that they undergo an electrocardiogram, echocardiography, and cardiology consultation within 2-4 months of surgery to evaluate left ventricular structure and ejection fraction [2]. Left ventricular hypertrophy (LVH) is a common finding in this patient population and is likely related to the severity of GAA repeats [3]. A study by Norrish et al. showed that hypertrophic cardiomyopathy is present in up to 85% of patients with FRDA [4]. Patients having bulbar symptoms (drooling, slurred speech, and choking) are at high risk for aspiration and peri- and postoperative respiratory complications. This risk is compounded in FRDA patients with thoracic kyphoscoliosis, due to restrictive respiratory effects [5].

The increased incidence of cardiomyopathy, diabetes mellitus, and compromised respiratory function in these patients makes anesthetic management a challenging issue [1]. No clinical guidelines are established on general or regional anesthetic management of patients with FRDA [5], but regional and local anesthesia have been reported in several cases without complications [6-9]. Case reports have also shown successful general anesthesia using remifentanil with propofol, alfentanil with propofol, and isoflurane. Muscle relaxant use in patients with FRDA has been reported with varying results. One report showed hypersensitivity to tubocurarine, while others reported relatively normal responses to various non-depolarizing neuromuscular blocking agents (NMBAs), including tubocurarine, atracurium, and vecuronium [2]. Depolarizing neuromuscular blocking agents, such as succinylcholine, should be avoided in these patients due to the increased risk of hyperkalemia. This increased risk is thought to be due to the upregulation of postsynaptic acetylcholine receptors in patients with movement disorders [5,10-12].

Overall, patients with FRDA present a challenge to the anesthesiologist that can have serious consequences. This is particularly related to their abnormal cardiopulmonary physiology and multifactorial risk of respiratory failure from muscle weakness, higher incidence of OSA and scoliosis, and increased propensity for aspiration [1]. To maintain pace and provide safe care with the evolving landscape of both anesthesia and surgery, careful planning is warranted.

Here, we present a case of a 59-year-old male with a 28-year history of FRDA, with significant mobility limitations, presenting for a robotic-assisted right partial nephrectomy for a right renal mass. We discuss the challenges of robotic surgery as they pertain specifically to FRDA. Additionally, we explain our rationale for using our ERAS protocol, including the utilization of bilateral quadratus lumborum (QL) nerve blocks to limit the detrimental effects of opioids in an FRDA patient who is more susceptible to respiratory complications. We also discuss the safe use of sugammadex in patients with neuromuscular conditions including FRDA. In our case, our patient required no postoperative opioids and was discharged home ahead of schedule.

## Case presentation

**Figure 1:**
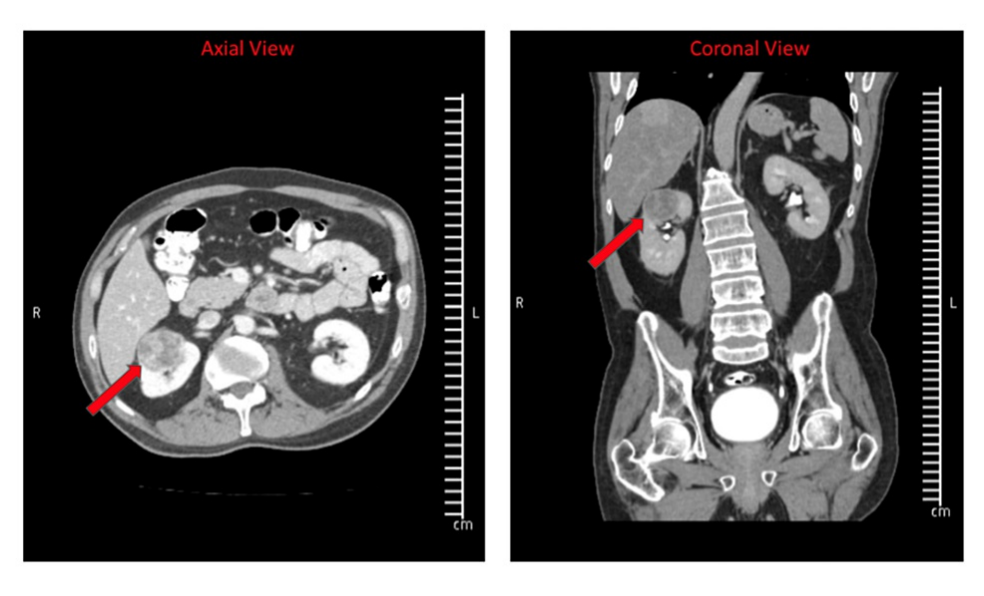
Axial and Coronal Computed Tomography Images of the Right Upper Pole Renal Mass (Arrows)

A 59-year-old male with a history of Friedreich ataxia, hypertension, non-Hodgkin’s lymphoma treated with chemotherapy and radiation in remission, type 2 diabetes mellitus, prior deep venous thrombosis no longer on treatment, and gastroesophageal reflux presented for robotic-assisted partial nephrectomy. The patient was diagnosed with FRDA 28 years prior to presentation for surgery and has one first-degree sibling who also has the disease. He had partial contractures of the upper and lower extremities requiring a motorized wheelchair for mobilization. The patient did also note a history of scoliosis, a common finding in patients with FRDA. This was treated as a child with a brace. The patient was 172.7 cm (5’8’’) and 68.6 kg (151 pounds) with a body mass index of 23 kg/m^2^. He participated in routine weekly physical therapy. The patient was referred to urology for the workup of hematuria and a urinary tract infection. At that time, a renal/bladder ultrasound was notable for a solid right renal mass. The patient subsequently underwent a computed tomography urogram of the pelvis/abdomen, which showed a 3.9-cm right upper pole kidney mass (Figure 1), and he was referred for surgical removal. After the risks/benefits were discussed by the patient and the surgeon, he was scheduled to have a robotic-assisted partial right nephrectomy.

The patient was evaluated by the preadmissions department at our institution. Laboratory values were notable for mild anemia with a hematocrit of 39.8% and a slightly reduced sodium of 133 mmol/L. An electrocardiogram showed normal sinus rhythm with nonspecific T wave abnormalities. Due to the abnormal electrocardiogram and since patients with FRDA frequently have cardiac involvement, he was referred to cardiology for optimization and clearance prior to surgery. A transthoracic echocardiogram was performed showing a normal ejection fraction, mild mitral regurgitation, and trace pulmonary regurgitation. Cardiac clearance was obtained.

On the morning of surgery, the patient was evaluated by the anesthesia team. He was noted to be frail with spasticity in all extremities. He was partially contracted, although he did have some strength and movement. He reported being able to pivot and stand for brief periods of time but otherwise used a motorized wheelchair for mobilization. He did report a baseline weak voice and subjective dysphagia; however, a swallow study was found to be normal. The patient’s point-of-care fingerstick glucose was 175 mg/dL. The anesthetic plan was discussed with the patient and included the use of our Enhanced Recovery After Surgery (ERAS) protocol from the preoperative to the postoperative period, in addition to general anesthesia. The patient and surgeon were in agreement with the plan.

**Figure 2:**
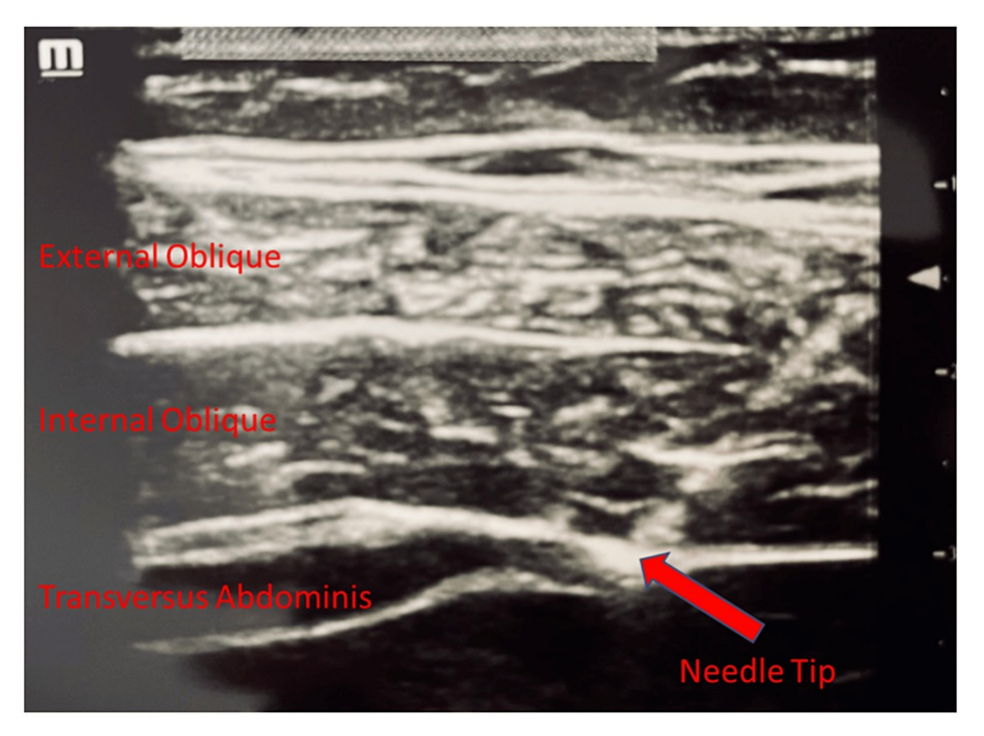
Ultrasound-Guided Quadratus Lumborum Block Using the QL1 Technique

The preoperative component of his anesthetic consisted of placing a scopolamine patch (1.5 mg) to prevent postoperative nausea and vomiting. Gabapentin 600 mg was administered orally. The patient was premedicated with 2 mg of intravenous midazolam for anxiolysis prior to performing QL blocks. The QL blocks were done under ultrasound guidance (Figure 2) via the QL1 method, which is the placement of a local anesthetic lateral to the quadratus lumborum muscle. In our technique, we used 10 mL of 1.33% liposomal bupivacaine, 10 mL of 0.5% bupivacaine, and 10 mL of normal saline, for a total of 30 mL to each side, for excellent local anesthesia spread. The patient was also hydrated with 750 cc of lactated ringers in the preoperative bay to aid in the prevention of hypotension associated with the induction of anesthesia.

The patient was then brought to the operating room, and monitors were applied. General anesthesia was induced with 150 mg propofol, 30 mg rocuronium, 60 mg lidocaine, and 100 mcg fentanyl. A 7.5-mm cuffed endotracheal tube was placed. We opted to preemptively place a second peripheral intravenous catheter due to the difficulty in reaching the patient’s extremities once positioned. The patient was then positioned in the flank position, right side up with an axillary roll placed.

Maintenance of anesthesia was achieved using a balanced anesthetic of 1.4%-1.6% sevoflurane and 50 mcg/kg/minute of propofol (3.45 mg/minute). In addition, the patient received 1,000 mg of intravenous acetaminophen, 4 mg of intravenous dexamethasone (a reduced dose from our standard ERAS protocol due to the patient’s diabetes mellitus history and marginal preoperative glucose level), and 4 mg of intravenous ondansetron as part of our multimodal regimen. He received a total of 0.6 mg of hydromorphone intraoperatively during the 2 ½ hours of surgical procedure time for comfortability while under anesthesia. The patient’s train of four (TOF) was monitored closely through the duration of the case, aiming for 3-4 with fade using a standard twitch monitor, along with a bispectral index (BIS) level target of 20-30.

The intraoperative course was notable for difficulty in freely moving the robotic arm closest to the patient’s right upper extremity. The placement of the patient’s arm to accommodate the contracture in the upper extremity was noted to be causing restriction. Adjustments to the patient’s position, while still maintaining the natural contracture, combined with adjustments to the robotic arm corrected the issue. The remainder of the surgical and anesthetic courses were uneventful.

Propofol was stopped when the closing process began, approximately 45 minutes before extubation. The patient was given 200 mg of sugammadex for the reversal of neuromuscular blockade. The patient had a return of TOF to 4/4 twitches. He followed commands and exhibited sustained head lift and hand grip with appropriate tidal volumes. Extubation was unremarkable. The patient was then placed on a standard oxygen mask and transported uneventfully to the recovery room.

The patient had no pain in the recovery room and did not require any opioids. Postoperatively, he received 1,000 mg intravenous acetaminophen every six hours for four doses as well as 600 mg of gabapentin before bed on the evening of surgery. The patient had low pain scores throughout his stay, requiring only three doses of intravenous ketorolac and no opioids. The patient was discharged home on postoperative day 1 rather than his planned discharge date of postoperative day two.

## Discussion

Robotic surgery has shown to have numerous benefits over traditional and laparoscopic surgery, namely, superior precision and accuracy, as the robotic system functions much similar to a human wrist, allowing for small, concise movements. In fact, data shows that robotic surgery has fewer instances of conversion to laparotomy compared to laparoscopic. By the nature of avoiding the open laparotomy technique, hospital stays are shorter, and patients require fewer blood transfusions. Robotic surgery is beneficial in settings in which space can be limited given the improved range of movement compared to laparoscopic surgery. As such, robotic-assisted urologic procedures in the confined space of the pelvis have gained increasing popularity [13].

However, robotic surgery comes with a number of matters that must be addressed by the anesthesia team. Positioning is of the utmost importance to allow for docking the robotic system safely as well as the optimization of surgical conditions to allow for the best possible outcomes. The steep Trendelenburg position used in many robotic surgeries, with the associated upward movement of the diaphragm into the thoracic cavity, is not without consequences in regard to hemodynamics. When positioning is combined with the presence of pneumoperitoneum, profound physiologic changes can occur, including reduced lung compliance and functional residual capacity, pulmonary edema, and ventilation-perfusion mismatch. Additionally, the gravity dependence related to the positioning can lead to airway and/or cerebral edema as well as vision loss. Fluid management and the likelihood of edema must be considered in the greater picture of the patient’s hemodynamics and can be challenging. Careful, advanced planning for robotic surgery is of the utmost importance as easy accessibility to the patient is often precluded by bulky arms and table positioning. Shifting of the patient’s torso during surgery can lead to undesirable stretch on the secured upper and lower extremities, leading to nerve damage [13].

Robotic surgery comes with a unique set of challenges to patients with neuromuscular conditions such as FRDA that have not yet been discussed in the literature. The cardiopulmonary pathophysiologies often seen in patients include structural heart conditions such as enlarged septums and LVH and restrictive lung processes from scoliosis and pulmonary disease related to OSA [1]. These abnormalities likely render the patient less likely to tolerate the physiologic changes seen with the use of the steep Trendelenburg position and the use of pneumoperitoneum required for robotic surgery. As such, planning for the patient’s anesthetic in the preoperative setting is paramount. Consideration regarding the need for additional intravenous access or more comprehensive monitors, such as arterial lines, should be determined in advance of the operating room.

In addition to physiologic changes seen with robotic surgery, positioning for surgery may have an added level of difficulty in patients with FRDA, particularly those that are partially or fully contracted. In our case, the surgeon had difficulty manipulating the arms of the robot, requiring adjustment of both the patient and the robot to allow the patient to safely undergo surgery. We recommend carefully tailoring the padding to maintain the patient’s natural position to avoid the robotic arms injuring the patient or neuropathic damage.

Perioperative pain control is an important component of all patient’s anesthetics. Those with FRDA can be particularly susceptible to the negative effects of opioids due to the underlying muscle weakness associated with their disease. Higher prevalence of scoliosis and OSA also place the patients in a vulnerable place in regard to their respiratory status in the intraoperative and postoperative respiratory status. To the best of our knowledge, no literature exists on the use of multimodal opioid-sparing ERAS protocols in FRDA patients or in the broader neuromuscular patient population.

Many institutions have put into place ERAS protocols for a myriad of surgery types, including colorectal, spine, bariatric, esophageal, and gynecology oncology, among others. ERAS protocols typically involve all phases of a patient’s care, including preadmissions through discharge to home, and aim to reduce the length of hospital stays and reduce patient complications and readmission rates [14]. Depending on the type of surgery and the protocol used, various aspects of the patient’s care are closely managed with the rationale that many small factors will add up to an overall improved outcome. Optimization of nutrition status may begin several weeks before surgery. Opioid-sparing multimodal analgesia, to reduce untoward effects of perioperative opioids such as reduced bowel motility and respiratory depression, are the mainstays of ERAS protocols. Reduction in opioid administration is often achieved using non-opioid oral and intravenous analgesics, regional nerve blocks, epidural catheters, etc. Additionally, best fluid management techniques are implemented based on the surgical type and patient factors. Nausea and vomiting prevention and treatment are key components of ERAS protocols as well [15]. Evidence that ERAS protocols improve patient outcomes and experiences and reduce hospital lengths of stay are promising. Ashok et al. reported that ERAS protocols improve outcomes and reduce the length of hospital stays for patients undergoing esophageal surgery [16]. Likewise, Zhou et al. have stated that ERAS protocols have lessened hospital lengths of stay without increasing patient complications, rates of readmission, need for reoperation, or visits to the emergency room following surgery in bariatric surgery. Additionally, rates of postoperative nausea and vomiting were reduced, while the overall patient recovery was enhanced [17]. Importantly, studies exist with evidence that ERAS protocols improve care in urologic surgery. A study by Brooks et al. showed improvement in quality of life in early recovery in urologic surgery with ERAS as well as an improved hospital financial footprint with the use of ERAS in patients undergoing radical cystectomy [18].

We opted to use a multimodal opioid-sparing anesthetic for our patient to reduce the need for intraoperative and postoperative opioid analgesia. We also felt strongly that the multimodal prevention of postoperative nausea and vomiting plays a key role in the optimization of recovery following surgery. We administered gabapentin and placed a scopolamine patch preoperatively. We also opted for preoperative bilateral ultrasound-guided QL nerve blocks, as the literature suggests their superiority in analgesia over more traditional nerve blocks, such as transversus abdominis plane (TAP) blocks. In a study comparing TAP blocks to QL blocks in inguinal hernia surgery, there was a lower mean dose of analgesia required in the QL group and an increase in time to the first rescue dose needed [19]. Likewise, a randomized controlled study by Huang et al. showed reduced cumulative morphine patient-controlled analgesia consumption at six, eight, 12, 36, and 48 hours in patients receiving QL over TAP block in laparoscopic colorectal surgery [20]. Studies in various other types of surgeries consistently show superiority of the QL block over the TAP block. Additionally, QL blocks have been used as part of ERAS protocols for robotic urologic surgeries [21]. Our patient required a total of 100 mcg of fentanyl and 0.6 mg of hydromorphone intraoperatively to maintain comfort under anesthesia. Postoperatively, the patient required no opioids in the recovery room. He was maintained on intravenous acetaminophen every six hours standing. For the duration of his recovery in the hospital, he required only three intravenous doses of ketorolac and did not require any opioids. Given the lack of opioids following a substantial surgery, we believe the QL blocks had excellent opioid-sparing effects.

Based on the success in regard to reduced opioid consumption and overall easy recovery for our patient, we recommend the consideration of the use of ERAS protocols in all patients with neuromuscular diseases. We suggest the benefits of regional and neuraxial anesthesia, such as their opioid-sparing effects, be weighed against the risks of using these modalities in the setting of preexisting neurologic deficits. For instance, regional anesthesia has safely and successfully been used in the FRDA patient population [9]. On the other hand, literature on the use of neuraxial anesthesia for patients with multiple sclerosis is less clear, reflecting the importance of tailoring the anesthetic approach to each patient individually [22].

An important consideration when caring for a patient with FRDA is the use of neuromuscular blocking agents (NMBAs). Succinylcholine, a depolarizing NMBA, likely carries the risk of hyperkalemia and cardiac arrest in patients with FRDA, as it typically does in cases of muscular diseases [23]. Non-depolarizing NMBAs are typically considered safer for use in patients with FRDA; however, the literature is mixed regarding the increased sensitivity to these agents. Most clinicians err on the side of caution with non-depolarizing NMBAs in those with neuromuscular disorders, due to the high prevalence of increased sensitivity seen, such as in Duchenne muscular dystrophy and myasthenia gravis. However, specifically in the FRDA patient population, there are a number of articles discussing the safe use of non-depolarizing NMBAs with responses on course to their non-FRDA counterparts [24]. A study by Mouloudi et al. yielded a normal response to atracurium in FRDA patients [25]. Likewise, a study by Schmitt et al. showed that the duration of the clinical action of rocuronium was comparable between patients with FRDA and those without the disease [11]. Nonetheless, in many cases of FRDA, muscle strength is often compromised by the disease process itself, and the use of TOF monitoring can significantly help guide the administration of NMBAs.

The reversal of NMBAs is critically important in patients with underlying muscular disease as residual neuromuscular weakness can render them highly susceptible to respiratory failure. Sugammadex is a 𝛾-cyclodextrin agent that binds and removes steroidal-based non-depolarizing NMBAs from the circulation and excretes the bound complexes in the urine [26]. Many studies show the superiority of sugammadex over traditional agents such as neostigmine. The Sugammadex versus Neostigmine for Reversal of Neuromuscular Blockade and Postoperative Pulmonary Complications (STRONGER) study showed a 30% reduction in pulmonary complications with the use of sugammadex over neostigmine [27]. Likewise, Hristovska et al. showed that the reversal of rocuronium was faster with sugammadex over neostigmine for all levels of blockade [28]. Gurunathan et al. concluded that sugammadex is successful in reversing steroidal non-depolarizing NMBAs in patients with neuromuscular disorders [29]. While the literature is scarce regarding the use of sugammadex specifically in FRDA patients, there is substantial literature to suggest its safe and adventitious use in the neuromuscular disease population as a whole. As such, we opted to use sugammadex for reversal in our patient. After administering 200 mg of intravenous sugammadex, the patient’s TOF was 4/4. The patient exhibited favorable signs of extubation, including following commands, sustained head lift and hand grip, and appropriate tidal volumes. The patient’s extubation was straightforward, and postoperatively, he did not exhibit any signs of residual weakness. In fact, the patient was back to his baseline strength on postoperative day 1 and was discharged early on this basis. We attribute his early discharge primarily to the opioid-sparing effects of our multimodal analgesia along with full neuromuscular recovery after surgery. As such, we recommend that clinicians consider the use of sugammadex over neostigmine in patients suffering from FRDA.

While our patient had what we considered to be an excellent outcome, there are two limitations to our report. Our patient had partial contractures of his extremities, which needed to be addressed after initially positioning the patient. In the future, consideration can be given to using adjunctive preoperative muscle relaxants. A study by Bhatia and Buvanendran showed promising results with their use, as part of Rush University’s ERAS protocol, which may aid in the improved range of motion during surgery. This may reduce the incidence of complications related to positioning [30]. Additionally, adjunct medications of this class have shown to have the added benefit of lower pain intensity, reduced analgesia needs, and reduced length of post-anesthesia care unit (PACU) stay following laparoscopic surgery [31]. Given the possibility of increased issues with positioning for robotic surgery, we advocate for weighing the risk/benefit of these agents in the preoperative setting ahead of surgery. Additionally, we used standard (TOF) monitoring to assess the depth of neuromuscular blockade in this patient. However, TOF monitoring is subjective, and studies have shown that when the TOF ratio is greater than 0.4, the absence of fade (when all four responses to TOF stimulation appear equal) is not a reliable indicator of adequate neuromuscular blockade reversal [32]. Moving forward, we will aim to obtain quantitative TOF monitors for our institution.

## Conclusions

Here, we present our 59-year-old patient with a long-standing history of FRDA with resultant mobility restrictions presenting to our institution for the resection of a right renal mass via robotic-assisted right partial nephrectomy. Preoperatively, we instituted our ERAS protocol, including the use of gabapentin, scopolamine patch, and bilateral ultrasound-guided quadratus lumborum nerve blocks. Intraoperatively, we used a balanced anesthetic of sevoflurane and propofol along with intravenous acetaminophen, dexamethasone, and ondansetron. The intraoperative course was notable for difficulty in the positioning of the patient due to contractures related to the patient’s FRDA. Additional padding and manipulation of the robotic arms resolved the issue. The patient received sugammadex for the reversal at the end of the case with excellent recovery of neuromuscular function. Postoperatively, he received scheduled intravenous acetaminophen. He required no postoperative opioids and was discharged home a day earlier than anticipated as he was noted to be back to his baseline strength.

As novel advances in surgery and anesthesia arise, new concerns and improvements also come to light. These changes must be addressed, particularly in our most vulnerable patient populations who are already at an increased risk from surgery and anesthesia, such as those with FRDA. The literature has lagged behind these changes in regard to the best anesthesia care. Robotic surgery poses an increased challenge due to the changes in hemodynamics from positioning superimposed on the pathophysiologic cardiopulmonary abnormalities seen with FRDA. Preoperative preparation is of utmost importance to predict and manage complications associated with these changes. As such, we recommend preoperative planning for the need for advanced hemodynamic monitors such as arterial lines and additional intravenous access as the ability to reach the patient becomes difficult once the robot is docked. We also suggest tailoring the padding to meet the natural position of the patient to avoid neuropathic injury. The preoperative use of adjunct medications such as muscle relaxants should be considered. Additionally, ERAS protocols have become the mainstay of many types of surgery with the goal of optimizing nutrition and fluid management, reducing the need for opioids, and avoiding postoperative nausea and vomiting to allow for the easiest recovery possible. Literature is lacking regarding the use of these protocols in patients with neuromuscular diseases such as FRDA, although patients with these disorders may benefit the most from them. We advocate considering ERAS protocols in patients with neuromuscular disease and suggest a risk/benefit analysis be done on an individual basis. Moreover, sugammadex has consistently shown to be superior to standard neuromuscular reversal and safe in both the general patient population and those with neuromuscular disorders. However, specifically in regard to the sugammadex in patients with FRDA, the literature is scarce. We safely used sugammadex in our patient with straightforward extubation. As such, we recommend the clinician consider the use of sugammadex in patients with FRDA in the future. The combination of our multimodal opioid-sparing anesthetic technique and the use of sugammadex allowed our patient to have a recovery that required no opioids and to be discharged home ahead of schedule.
